# Comparison of high risk factors (hot food, hot beverage, alcohol, tobacco, and diet) of esophageal cancer: A protocol for a systematic review and meta-analysis

**DOI:** 10.1097/MD.0000000000015176

**Published:** 2019-04-26

**Authors:** Tianci Chai, Zhimin Shen, Peipei Zhang, Yuhan Lin, Sui Chen, Zhenyang Zhang, Wenwei Lin, Mingqiang Kang, Jiangbo Lin

**Affiliations:** a Department of Thoracic Surgery, Fujian Medical University Union Hospital; b The Graduate School of Fujian Medical University; c School of Stomatology, Fujian Medical University, Fuzhou, China.

**Keywords:** alcohol, diet, esophageal cancer, hot beverage, hot food, tobacco

## Abstract

**Background:**

Esophageal cancer (EC) is one of the most common malignant tumors with a poor prognosis and identified as one of the leading causes of cancer death in the world. Many studies have reported that the incidence of EC is closely related to the intake of alcohol, hot food, and hot beverages, as well as smoking and diet. However, there is a lack of studies on the quantitative analysis of these risk factors for EC. If the solid quantitative evidence of these risk factors is provided for the prevention of EC, the prevalence of EC can be effectively reduced. We will conduct a systematic review and meta-analysis of high risk factors for EC in order to provide reliable evidence for the prevention of EC.

**Methods and analysis:**

We will search PubMed (Medline), the Cochrane Central Register of Controlled Trials, Embase, and Google Scholar for related studies published without language restrictions before December 1, 2019. Two review authors will search and assess relevant studies independently. Trials used a case-control, cross-sectional, cohort studies, randomized controlled trials (RCTs), and quasi-RCTs will be included. We will perform subgroup analysis in sex, age, ethnicity, and region.

**Results:**

The results of this study will be published in a peer-reviewed journal.

**Conclusion:**

We will perform a systematic review and meta-analysis of high risk factors for EC in order to provide reliable evidence for the prevention of EC. However, because of the characteristics of disease and intervention, large-sample trials that meet the inclusion criteria of this study may be insufficient. We will consider including some high-quality small-sample related trials, which may lead to high heterogeneity and affect the reliability of the results.

## 1 Introduction

Esophageal cancer (EC) is one of the most common malignant tumors with a poor prognosis and identified as one of the leading causes of cancer death in the world.^[1,2]^ Although EC is rare in most western countries, its incidence varies widely around the world, and is relatively high in Asia, southern and eastern Africa, and northwestern France, where the incidence of EC exceeds 100 per 100,000 persons per year.^[3]^ This malignant tumor mainly consists of 2 main histological types, esophageal squamous cell carcinoma (ESCC) and esophageal adenocarcinoma (EAC), with different etiology and pathological characteristics, among which ESCC is the main one.^[4]^

The risk factors for EC follow a geographic pattern. In western countries with a low incidence of EC, smoking and drinking are the main risk factors for the occurrence of EC,^[5–10]^ while in regions with a high incidence, the consumption of food and beverage with high temperature and the diet of low fruit and vegetable intake is closely related to the occurrence of EC.^[11–14]^

The correlation between these high risk factors and the incidence of EC is of great clinical significance. For malignant tumors with poor prognosis, these risk factors can be changed and can be easily eliminated, with little impact on patients, thus effectively reducing the incidence of EC. We will conduct a systematic review and meta-analysis of high risk factors for EC in order to provide reliable evidence for the prevention of EC.

## 2 Objective

We will evaluate the high risk factors (hot food, hot beverage, alcohol, tobacco, and diet) of EC among cases and controls.

## 3 Methods

This protocol is conducted according to the Preferred Reporting Items for Systematic Review and Meta-Analysis Protocols (PRISMA-P) statement.^[15]^ We will report the results of this systematic review and meta-analysis adhere to the Preferred Reporting Items for Systematic Reviews and Meta-Analyse (PRISMA) guidelines.^[16]^ This protocol has been registered in the PROSPERO network (registration number: CRD42019124789).

### 3.1 Eligibility criteria

#### 3.1.1 Types of studies

Trials used a case-control, cross-sectional, cohort studies, randomized controlled trials (RCTs), and quasi-RCTs published or unpublished will be included, which have been completed and compared the correlation between these risk factors (hot food, hot beverage, alcohol, tobacco, and diet) and the incidence of EC.

#### 3.1.2 Types of participants

The participants will be adults diagnosed with EC histologically or cytologically confirmed. There will be no restrictions on sex, ethnicity, economic status, and education.

#### 3.1.3 Types of exposure factors

According to the types of exposure factors for patients with EC, the studies included will be divided into the following categories.

- Studies examined the consumption of hot food and beverage among EC cases and controls.
- Studies examined the consumption of alcohol among EC cases and controls.
- Studies examined the tobacco among EC cases and controls.
- Studies examined the diet among EC cases and controls.

#### 3.1.4 Types of outcome measures

The outcome will be correlation between high risk factors and the incidence of EC.

### 3.2 Information sources

We will search PubMed (Medline), the Cochrane Central Register of Controlled Trials, Embase, and Google Scholar for related studies published without language restrictions before December 1, 2019 without language restrictions.

### 3.3 Search strategy

We will use the relevant keywords or subject terms adhered to Medical Subject Heading (MeSH) terms to search for eligible studies in the electronic databases which were mentioned above without language restrictions. The PubMed search strategies are shown in Table 1.

**Table 1:**
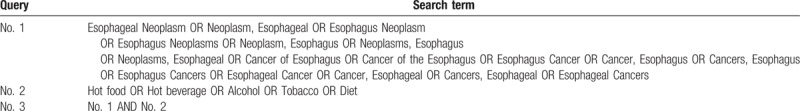
PubMed search strategies.

### 3.4 Data collection and analysis

We will utilize the measures described in the Cochrane Handbook for Systematic Reviews of Interventions to pool the evidence.^[17]^

#### 3.4.1 Study selection

Two reviewers (TCC, ZMS) will investigate each title and abstract of all literatures searched independently and identify whether the trials meet the inclusion criteria as designed and described in this protocol. Two authors (TCC, ZMS) will in duplicate and independently screen the full text of all potential eligible studies to exclude irrelevant studies or determine eligibility. The two reviewers will list all the studies included and document the primary reasons of exclusion for studies that do not conform to the inclusion criteria. Disagreements between the two authors will be resolved by discussing with the third author (JBL), if necessary, consulting with the fourth author (MQK). We will show the selection process in details in the PRISMA flow chart.

#### 3.4.2 Data extraction and management

The 2 authors (TCC, ZMS) will extract the following data independently from the studies included.

- Study characteristics and methodology: the first author, publication date, country or region, study design, periods of data collection, follow-up duration, total duration of study, and withdrawals, etc.
- Participant characteristics: sex, age, tumor stage, pathology diagnosis, ethnicity, performance status, pathologic tumor size, and inclusion criteria, etc.
- Interventions (exposure factors): hot food and drink intake (temperature scale), alcohol consumption, tobacco intake, dietary habits (food composition), etc.
- Other data: overall survival, 5-year survival, median survival, 95% confidence intervals (CIs), quality of life, adverse events, etc. We will record all the date extracted in a pre-designed table and consult the first author of the study by e-mail before determining eligibility, if the reported data of which are unclear or missing.

### 3.5 Assessment of risk of bias in included studies

Two authors (TCC, ZMS) will use the Cochrane Handbook for Systematic Reviews of Interventions to assess the risk of bias of each study included independently based on the following ranges: random sequence generation (selection bias); allocation concealment (selection bias); blinding of participants and personnel (performance bias); blinding of outcome assessment (detection bias); incomplete outcome data (attrition bias); selective outcome reporting (reporting bias); other bias.^[15]^

Each domain will be assessed as high, low, or uncertain risk of bias. The results and details of assessment will be reported on the risk of bias graph.

### 3.6 Data analysis

The data will be synthesised by Review Manager 5.3 software. We will conduct a systematic review and meta-analysis only if the data gathered from included trials are judged to be similar enough to ensure a result that is meaningful. The chi-squared test and *I*^2^ statistic will be used to assess statistical heterogeneity among the trials included in matched pairs comparison for standard meta-analysis. The random effect model will be applied to analyze the data, if there is substantial heterogeneity (*P* < .1 or *I*^2^ statistic >50%) and the trials will be regarded to be obvious heterogeneous. Otherwise, we will utilize fixed effect model to analyze the data. Mantel–Haenszel method will be adopted to pool of the binary data. The results will be reported in the form of relative risk (RR) between 95% CI of the date. The continuous data will be pooled by inverse variance analysis method and the results will be shown in the form of standardized mean difference (SMD) within 95% (CI) of the date.

#### 3.6.1 Subgroup analysis

If there is high heterogeneity and the data are sufficient, subgroup analysis will be conducted to search potential causes of heterogeneity. Subgroup analysis will be performed in ethnicity, history of smoking, tumor stage, and type of operation.

#### 3.6.2 Sensitivity analysis

Sensitivity analysis will be conducted to assess the reliability and robustness of the aggregation results via eliminating trials with high bias risk.

### 3.7 Publication bias

If there are ≥10 trials included, we will construct a funnel plot and use Egger test to assess publication bias. If reporting bias is suspected, we will consult the study author to get more information. If publication bias does exist, we will apply the fill and trim method to analyze publication bias in the trials.^[18]^

### 3.8 Evidence evaluation

We will evaluate all the evidence according to the criteria of GRADE (imprecision, study limitations, publication bias, consistency of effect, and indirectness bias). The quality of all evidence will be evaluated as 4 levels (high, moderate, low, and very low).^[19]^

## 4 Discussion

EC is one of the most common malignant tumors with a poor prognosis and identified as one of the leading causes of cancer death in the world. This malignant tumor mainly consists of 2 main histological types, esophageal squamous cell carcinoma (ESCC) and esophageal adenocarcinoma (EAC), with different etiology and pathological characteristics, among which ESCC is the main one.

In western countries with a low incidence of EC, smoking and drinking are the main risk factors for the occurrence of EC, while in regions with a high incidence, the consumption of food and beverage with high temperature and the diet of low fruit and vegetable intake is closely related to the occurrence of EC. The correlation between these high risk factors and the incidence of EC is of great clinical significance. For malignant tumors with poor prognosis, these risk factors can be changed and can be easily eliminated, with little impact on patients, thus effectively reducing the incidence of EC.

We will conduct a systematic review and meta-analysis of high risk factors for EC in order to provide reliable evidence for the prevention of EC. We will try to include some small sample studies. Although our team has experience in carrying out a systematic review and meta-analysis, there may be high heterogeneity and low reliability of evidence, which is the limitation of this study.

## Author contributions

Jiangbo Lin and Mingqiang Kang is the guarantor of the article. Tianci Chai and Zhimin Shen conceived and designed the study. Tianci Chai and Zhimin Shen drafted this protocol. Tianci Chai, Sui Chen, Zhenyang Zhang, Wenwei Lin, Peipei Zhang, and Yuhan Lin will perform the search, screening, and extraction. Jiangbo Lin and Mingqiang Kang have strictly reviewed this protocol and approved of publication. Tianci Chai and Zhimin Shen contributed equally to this work.

**Conceptualization:** Zhimin Shen, Sui Chen, Zhenyang Zhang, Jiangbo Lin.

**Data curation:** Tianci Chai, Zhimin Shen, Peipei Zhang, Wenwei Lin.

**Formal analysis:** Tianci Chai, Yuhan Lin, Wenwei Lin.

**Funding acquisition:** Zhenyang Zhang, Jiangbo Lin.

**Investigation:** Tianci Chai.

**Methodology:** Tianci Chai, Zhimin Shen, Yuhan Lin, Sui Chen, Mingqiang Kang.

**Project administration:** Tianci Chai, Sui Chen.

**Resources:** Tianci Chai, Jiangbo Lin.

**Software:** Tianci Chai, Zhimin Shen.

**Supervision:** Yuhan Lin, Mingqiang Kang, Jiangbo Lin.

**Validation:** Zhimin Shen, Peipei Zhang, Yuhan Lin.

**Visualization:** Zhimin Shen, Peipei Zhang, Yuhan Lin, Mingqiang Kang, Jiangbo Lin.

**Writing – original draft:** Tianci Chai, Peipei Zhang, Zhenyang Zhang.

**Writing – review & editing:** Tianci Chai, Zhimin Shen, Zhenyang Zhang, Mingqiang Kang.
